# Primary pulmonary lymphoma in children

**DOI:** 10.1186/s13023-019-1009-5

**Published:** 2019-02-08

**Authors:** Xiaohui Wu, Chunju Zhou, Ling Jin, Hui Liu, Jinrong Liu, Shunying Zhao

**Affiliations:** 1 Department of Respiratory Medicine, Beijing Children’s Hospital, Capital Medical University, Nanlishi Road 56, Xicheng District, Beijing, China; 2 Department of Pathology, Beijing Children’s Hospital, Capital Medical University, Nanlishi Road 56, Xicheng District, Beijing, China; 3 Hematology Oncology Center, Beijing Children’s Hospital, Capital Medical University, Nanlishi Road 56, Xicheng District, Beijing, China

**Keywords:** Primary pulmonary lymphoma, Children, Immunodeficiency

## Abstract

**Background:**

Primary pulmonary lymphoma (PPL) is a rare disease, especially in children. We analyse the clinical features of PPL in 4 children to strengthen a understanding of it.

**Results:**

We reported a case series of 4 pediatric patients with PPLs including three diffuse large B-cell lymphomas and one natural killer-T cell lymphoma. All patients presented with unknown fever and cough as well as weight loss and fatigue. The white blood cell count was reduced in three patients and increased in the other one. The level of C-reactive protein was increased in all patients. The procalcitonin concentrations and bone marrow specimens were normal. Multiple or single pulmonary nodules with halo signs were found in all patients and air bronchograms found in 3 of them on chest computed tomography scan. Primary immunodeficiency was diagnosed in two patients who was performed genetic analysis.

**Conclusions:**

When a patient presents with long-term fever, high C-reactive protein level, leukopenia/leukocytosis, and multiple or single pulmonary nodules with a “halo sign” and air bronchogram on computed tomography, a possibility of PPL should be considered. A co-existance of immunodeficiency needs to be further investigated in patients with PPL.

## Background

Malignant lymphomas are proliferative diseases of lymphoid tissue and are classified as Hodgkin lymphoma (HL) and non-Hodgkin lymphoma (NHL). Pulmonary lymphomas can be classified as primary (PPL) and secondary pulmonary lymphomas (SPL). Pulmonary lymphomas is defined as primary when they affect one or both lungs without any evidence of extra-pulmonary involvement for at least 3 months following the diagnosis. An exception to these criteria is when the lungs are the principal site of involvement (patients with satellite nodes can be considered to have PPL). PPL is rare, accounting for only 0.5–1% of primary pulmonary malignancies [1]. It most commonly occurs in adults (median age 60 years) and is particularly rare in children [2]. Here, we analyse clinical features of PPL to strengthen a better understanding of it.

## Patients and methods

Four patients were diagnosed with PPL between January 2009 and December 2017 in Department of Respiratory Medicine of Beijing Children’s Hospital. All diagnoses were made by positive pathological findings of lung biopsies obtained surgically.

Data collected in this retrospective study included patient age, gender, main symptoms, medical history, chest X-ray film, computed tomography (CT) scan, results of bone marrow biopsy, laboratory testing (blood counts, C-reactive protein [CRP] and procalcitonin [PCT] concentrations), and pathological results.

## Results

### Clinical features

Four patients had a median age of 8 years (range, 5–11 years) and comprised three boys and one girl. All patients have been misdiagnosed having pneumonia and had received anti-bacterial, anti-tuberculosis or anti-fungal treatment before the correct diagnosis was made. The mean interval between onset of symptoms and final diagnosis was 57 days (range 28–81 days). Four patients presented with fever and cough. Three patients had persistent high fever for more than 1 month, and one patient (No.1) had intermittent fever. All four patients had mild cough, weight loss and fatigue.

### Laboratory investigations

The white blood cell count was reduced in three patients and increased in the other one. One patient had a slightly increased CRP level and the other three patients had remarkably increased CRP of greater than 100 mg/L (the normal range is 0-8 mg/L). PCT concentrations were normal. All patients received bone marrow puncture and no abnormalities were found by morphological, immunological and cytological examinations. A bronchoalveolar lavage was performed in 3 of 4 patients (Patient No. 2, 3 and 4). Neither the cytological or the microbiological examinations of bronchoalveolar lavage fluid had positive findings.

### Imaging manifestations

**Fig. 1:**
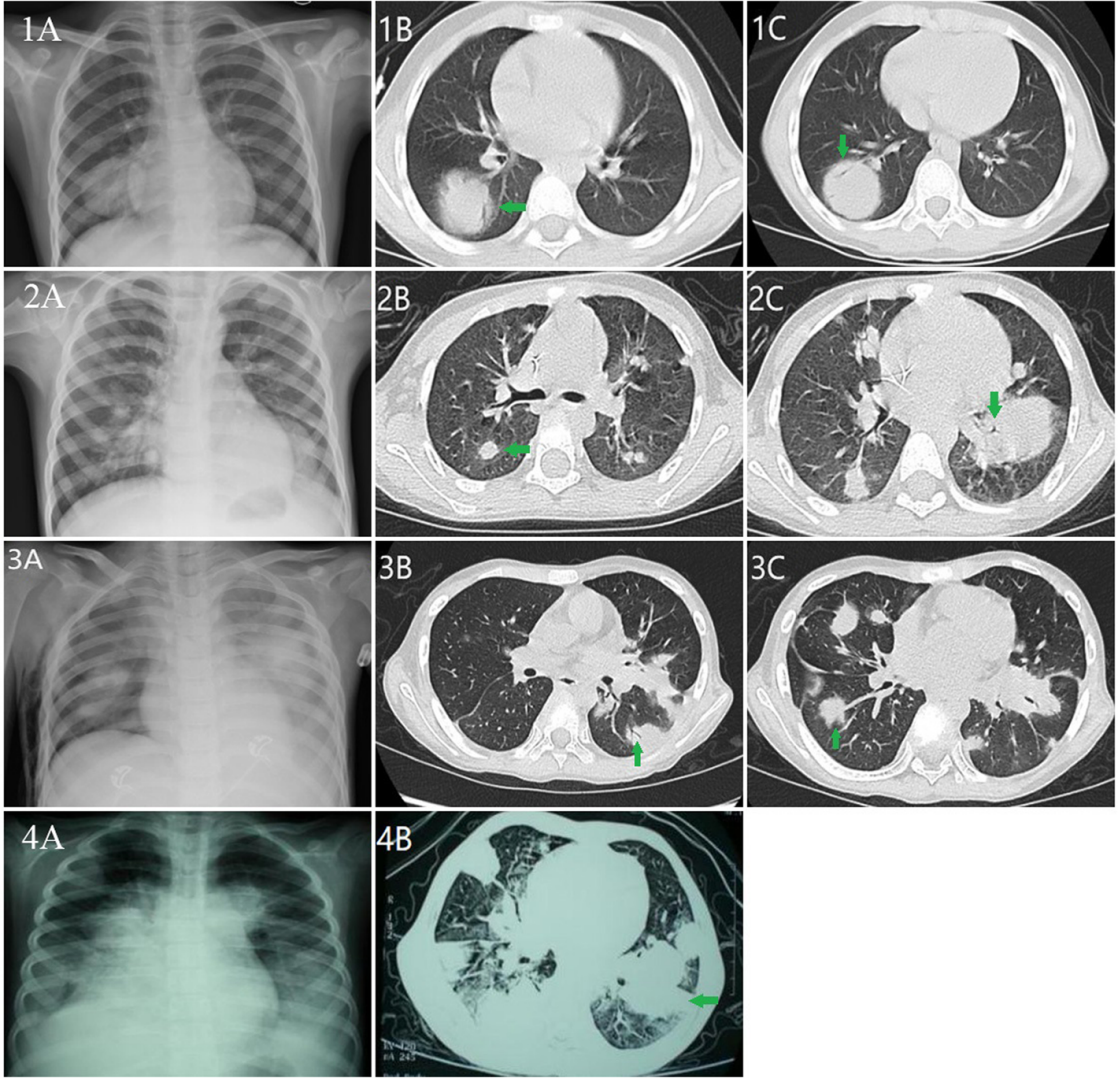
Chest film and CT scan shows a circular consolidation (3.7 × 3.1 cm) with poorly defined borders in the right lower lobe. An air bronchogram is readily seen (1a-c, Patient 1); Chest film and CT image shows multiple nodular masslike areas of consolidation with halo signs and air bronchograms in both lungs (2a-c, Patient 2 and 3a-c, Patient 3); Chest film and CT image shows multiple nodular masslike areas of consolidation with halo signs in both lungs (4a-b, Patient 4)

**Fig. 2:**
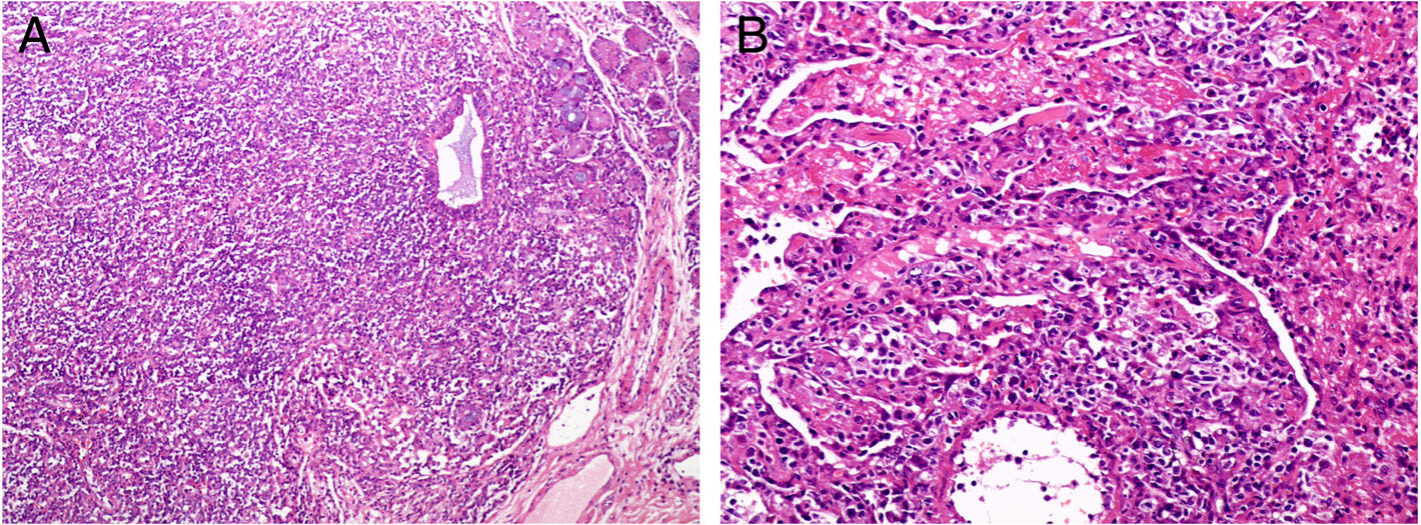
The lesions showed extensive infiltration of atypical lymphlid cells. **a** Patient 1: DLBCL (HE,× 100); **b** Patient 3: NK/T-cell lymphoma (HE,× 200)

All patients underwent chest radiographs and CT scans. Patient 1 had a single pulmonary mass (3.7 × 3.1 cm) with air bronchograms (Fig. 1, 1A, 1B, 1C) whereas the remaining three patients had multiple nodules/masses (Fig. 12A,2B,2C,3A,3B,3C,4A,4B), with air bronchograms in two of them. All consolidated lesions had halo sign around.

### Pathological findings

Pathological diagnoses were established by examination of biopsies obtained by open thoracotomy in three patients and thoracoscopy in one (Patient No.4). All patients were diagnosed as NHL, 3 patients had DLBCLs and one patients had extranodal natural killer (NK)-T cell lymphoma, nasal type (Fig. 2).

### Underlying disease

Two patients were diagnosed with immunodeficiency. Patient No.1 had experienced frequent sinopulmonary infections starting from infancy, and the laboratory test showed a significant decrease in immunoglobulin (Ig) A and CD4 + cells (IgA < 0.0667 g/L,CD4 + cells 6.1%). And the genetic tests (Next-Generation Sequencing technology) showed I Ligase IV syndrome. No abnormality in Igs and T-lymphocyte Subset was found in Patient No.3, but this boy had a history of recurrent respiratory infections, and his genetic examination showed immunodeficiency-21. Patient No.2 refused to take genetic test after the diagnosis of PPL. Patient No.4 did not receive genetic testing because the genetic testing technology was not widely used at the time of diagnosis.

### Treatment and outcome

Patient No.3 diagnosed as extranodal NK-T cell lymphoma, nasal type received chemotherapy with SMILE regimen (Dexamethasone, Methotrexate, Ifosfamide, L-asparaginase, Etoposide). He achieved partial remission after the first course, but he got fever again and his pulmonary lesions progressed rapidly during the second course. Unfortunately, the boy died of respiratory failure after two chemotherapy sessions. The other three patients refused further treatment after diagnosis of PPL and died.

Clinical characteristics of 4 patients are shown in Table 1.

**Table 1:** Clinical features and laboratory features, genetic evaluation and prognosis of 4 patients with PPL

	Patient 1	Patient 2	Patient 3	Patient 4
Gender	Male	Male	Male	Female
Age	7-years-5-months	5-years-2-months	11-years-1-month	8-years-4-months
Presentation	Intermittent fever, cough	Persistent fever,cough	Persistent fever	Persistent fever
Physical examination	Normal	Normal	Normal	Normal
Past history	History of recurrent pneumonia (At the age of 4 months, 2-years-old, 3-years-old, 6-years-old and 7-years-old)	Normal	History of recurrent respiratory infections (8–10 times per year)	Normal
Underlying disease	I Ligase IV syndrome	Unclear	Immunodeficiency-21	Unclear
Prognosis	Died	Died	Died of respiratory failure after two chemotherapy sessions	Died
WBC(× 10^9^/L)	1.1~6.4	2.1~7.8	2.5~6.7	4.3~8.8
CRP(mg/L)	73~168	5~23	20~118	120~180
PCT (ng/ml)	Normal	Normal	Normal	Normal
Pathological findings	Diffuse large B-cell lymphoma	Diffuse large B-cell lymphoma	NK/T-cell lymphoma	Diffuse large B-cell lymphoma
Immune globulin	IgA < 0.0067 g/L, IgG11.6 g/L, IgM1.12 g/L, IgE0.11 IU/ml	Normal	Normal	Normal
Lymphocyte subsets	NK cells (44.9%), B cell (30.9%); CD4+ cells (6.1%), CD8+ cells (19.6%)	Normal	Normal	Normal
ANA,dsDNA,ACA,ANCA	Normal	Normal	Normal	Normal
Bone marrow examination	Normal	Normal	Normal	Normal
Diagnostic procedure	Open lung biopsy	Open lung biopsy	Open lung biopsy	Thoracscopy lung biopsy
Cytological results of bronchoalveolar lavage fluid	–	A great number of neutrophils; A small amounts of phagocytes, epithelial cells and lymphocytes;	A great number of neutrophils, A small amounts of histiocytes, epithelial cells and lymphocytes;	A great number of red blood cells and neutrophis; A small amounts of phagocytes, epithelial cells and lymphocytes
Microbiological results of bronchoalveolar lavage fluid	–	Negative	*Streptococcus viridans*	Negative

*Abbreviations*: *ANA* antinuclear antibody, *ACA* anticardiolipin antibody, *ANCA* antineutrophil cytoplasmic antibody, *NK* natural killer

## Discussion

We firstly reported PPL in children. There is difference in pathological type between children and adult. In adult studies, the most common type of PPL is mucosa-associated lymphoid tissue (MALT) lymphoma, an extranodal marginal zone lymphoma that accounts for 80–90% of PPL cases. DLBCL is the second most common type of PPL, and both MALT lymphoma and DLBCL are NHLs [3, 4]. However, DLBCL is the main pathological type in our study.

Patients with PPL may present with fever, cough, dyspnea, chest pain, hemoptysis, and other systemic symptoms (weight loss and fatigue) [4, 5]. In our series, all patients had fever, cough and high CRP concentrations, which were similar to bacterial and fungal infection. These symptoms were nonspecific and contributed little to the diagnosis of PPL.

The radiological manifestations of lymphoma in the lung can be classified into four patterns: nodular, pneumonic or alveolar, bronchovascular or lymphangitic, and miliary nodules [6]. There may be two or more types in the same patient. The most common pattern of pulmonary lymphoma is nodules. Multiple bilateral lung nodules with air bronchograms are the commonest findings in PPL [7]. In this study, all patients had nodules/masses, three patients had air bronchograms. In addition, halo signs were found in all patients. The halo sign is mainly associated with invasive aspergillosis, but may be present in PPL patient. It is proposed that the halo sign is caused by invasion of lymphoma cells into the blood vessels with resultant bleeding into the surrounding tissue [4], or infiltration of tumor cells into the surrounding normal lung tissue [1].

NHL is a predominant malignancy in a number of primary immunodeficiency diseases. The incidence of NHL in immunodeficient patients was 59%, and the incidence of immunodeficiency was 17.5% in NHL [8–10]. Although the mechnisms of the increased incidence of lymphoproliferative disease in immunodeficient patients are not fully understood, deterioration in immunoregulation, chronic antigenic stimulation, and tumor suppressor system dysregulation are thought to be the main explanation for it [7, 11, 12]. Two of the 4 patients in this study had immunodeficiency, suggesting that immunodeficiency disease need to be investigated in patients with PPL.

This study focuses on the diagnosis of PPL. All four patients in this study died, although one received chemotherapy. Treatment options and prognosis of PPL were still not clear.

## Conclusions

When a patient presents with long-term fever, high C-reactive protein concentrations, leukopenia/leukocytosis, and multiple or single pulmonary nodules with a “halo sign” and air bronchogram on CT scan, a possibility of PPL should be considered. The co-existance of immunodeficiency disease needs to be investigated in patients with PPL.

## Abbreviations

CRP: C-reactive protein
CT: Computed tomography
DLBCL: Diffuse large B-cell lymphoma
HL: Hodgkin lymphoma
Ig: Immunoglobulin
MALT: Mucosa-associated derived lymphoma
NHL: Non-Hodgkin lymphoma
PCT: Procalcitonin
PPL: Primary pulmonary lymphoma
SPL: Secondary pulmonary lymphomas
